# Prediction of Transformation Products of Monensin by Electrochemistry Compared to Microsomal Assay and Hydrolysis

**DOI:** 10.3390/molecules24152732

**Published:** 2019-07-27

**Authors:** Lisa Kotthoff, Jan Lisec, Tanja Schwerdtle, Matthias Koch

**Affiliations:** 1 Department of Analytical Chemistry and Reference Materials, Bundesanstalt für Materialforschung und -prüfung (BAM), Richard-Willstätter-Straße 11, 12489 Berlin, Germany; 2 Institute of Nutritional Science, University of Potsdam, Arthur-Scheunert-Allee 114-116, 14558 Nuthetal, Potsdam, Germany

**Keywords:** transformation products, monensin, veterinary drugs, electrochemistry, hydrolysis, LC/HRMS

## Abstract

The knowledge of transformation pathways and identification of transformation products (TPs) of veterinary drugs is important for animal health, food, and environmental matters. The active agent Monensin (MON) belongs to the ionophore antibiotics and is widely used as a veterinary drug against coccidiosis in broiler farming. However, no electrochemically (EC) generated TPs of MON have been described so far. In this study, the online coupling of EC and mass spectrometry (MS) was used for the generation of oxidative TPs. EC-conditions were optimized with respect to working electrode material, solvent, modifier, and potential polarity. Subsequent LC/HRMS (liquid chromatography/high resolution mass spectrometry) and MS/MS experiments were performed to identify the structures of derived TPs by a suspected target analysis. The obtained EC-results were compared to TPs observed in metabolism tests with microsomes and hydrolysis experiments of MON. Five previously undescribed TPs of MON were identified in our EC/MS based study and one TP, which was already known from literature and found by a microsomal assay, could be confirmed. Two and three further TPs were found as products in microsomal tests and following hydrolysis, respectively. We found decarboxylation, *O*-demethylation and acid-catalyzed ring-opening reactions to be the major mechanisms of MON transformation.

## 1. Introduction

Transformation products (TPs) are structurally diverse intermediates formed during the (bio)-degradation of organic compounds like drugs. The formation of TPs can be induced by different processes, for example during the biotransformation in living organisms or during environmental degradation [1]. The derived TPs are potentially more harmful or more persistent than their precursors [2,3], rendering TPs, of i.e., veterinary drugs, to be relevant residues in foodstuff or environmental matrices [1,4]. Veterinary drugs are used to cure and prevent animal diseases and are found as residues in animal products as well as in environmental matrices, due to incomplete biotransformation processes and the use of dung as crop plant fertilizer [2,5]. In the environment, degradation processes like photolysis, hydrolysis, or microbial processes take place whereby TPs are formed [6,7]. The identification of novel TPs in environmental or dung samples using non-targeted liquid chromatography/high resolution mass spectrometry (LC/HRMS) is difficult due to the low concentrations of the drug residues [8,9]. Therefore, TP generation is often simulated by different approaches [10,11,12]. One promising instrumental simulation method is based on the combination of electrochemistry (EC) and mass spectrometry [13,14,15]. The online-coupling enables the detection of stable TPs but also reactive or short-lived species. The substances can be oxidized or reduced depending on the potential polarity. EC can simulate a broad reaction spectrum of the sample without complex matrices, which enables a simplified identification of TPs. Among others, EC was used in drug metabolism studies showing that cytochrome P450 mediated metabolic reactions in the liver could be simulated [13,14,15]. Methods of different complexity are known to simulate biotransformations in the laboratory [10,11,12]. A commonly used method is the cell-free incubation with human (HLM) or rat liver microsomes (RLM), because most drugs are metabolized in the liver. Additionally, environmental processes can be mimicked on a laboratory scale. The degradation of veterinary drugs by hydrolysis is an important transformation pathway. The hydrolysis of substances is catalyzed by acidic (H^+^) or alkaline (OH^−^) conditions. The organisation for economic co-operation and development (OECD) published a guideline “Hydrolysis as a Function of pH” [16] for chemicals to investigate the hydrolysis behavior and to identify hydrolysis products. 

To this end, the identification of TPs is mostly achieved by LC/HRMS coupling [1,8]. The chromatographically separated TPs are analyzed, and a high accurate mass can be obtained. In a suspected-target analysis of the data, empirical formulas can be found, and structure validation can be obtained by investigating the MS/MS fragmentation pattern. 

**Figure 1:**
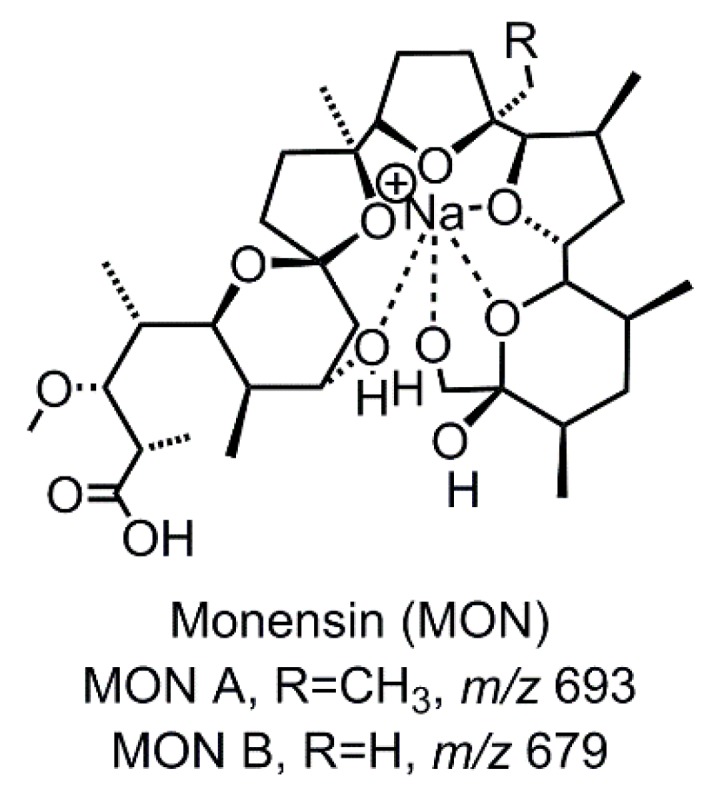
Structure of the investigated veterinary drug Monensin (MON). MON A is the main derivate, the amount of MON B is however significant.

Monensin (MON, Figure 1) is a widely used veterinary pharmaceutical with antibiotic and antimicrobial properties. It belongs to the group of ionophore antibiotics and is used as coccidiostat for poultry and cattle. Furthermore, MON shows potential as a growth promotor for cattle. MON has a polyether carboxylic structure and forms electrically neutral complexes with alkali metal cations. The MON-sodium complexes are very stable and due to the complexion, a quasi-cyclic conformation is formed.

Several studies were published investigating the transformation pathways of MON. Initially, metabolism investigations were made using microsome models [17,18] and the metabolites were also identified in manure [19,20,21]. Further studies on the degradation behavior of MON were conducted on pilot-scale composting [22], broiler litter [23], anaerobic digestion [24], degradation in soil [25], acid-catalyzed hydrolysis [26], and photolysis [27]. However, most of the studies focused on the degradation kinetics of MON and only to a lesser extent on the identification of TPs. A few studies investigated oxidative TPs, namely the conversion of MON with metalloporphyrins [28], the *Jacobson* catalyst [29] and the inhibition and biotransformation under different redox conditions [30].

In this study, electrochemistry was used for the first time to simulate oxidative TPs of MON and to compare the results with those from metabolism tests with RLM and hydrolysis experiments. The identification of partially new TPs by HRMS revealed the broad reaction spectrum of MON. In total, twelve TPs were identified, whereby nine showed different transformation reactions. EC leads to decarboxylation as major reaction, whereas O-demethylation was found by rat liver microsomes and acid catalyzed ring-opening by hydrolysis. All TPs extended the knowledge about MON’s properties. 

## 2. Results and Discussion

### 2.1. Electrochemical Investigation

A coupling between an electrochemical reactor (EC) and MS was used for the generation of TPs and their online identification by accurate mass determination. The MON-containing solution was pumped into an electrochemical flow-through cell where a potential was applied. The potential ranged from 0 up to 2500 mV and the reaction mix was transferred to the ESI-MS where the detection takes place. First, the experimental conditions were optimized. To this end, different types of working electrodes and mobile phases consisting of different solvent combinations and modifiers were tested (Table S1). Two working electrodes were used for the oxidation experiments, a glassy carbon (GC) electrode and a boron-doped-diamond (MD) electrode. The GC-electrode enabled the highest conversion rate of MON, leading to higher numbers of TPs. The best ionization of MON was achieved by using ammonium-based modifiers, where different concentrations of ammonium formiate were tested and the best results showed a concentration of 1 mM ammonium formiate. For use as a solvent, different combinations and ratios of methanol, acetonitrile (ACN) and water were tested. The increase of the ACN-ratio leads to less intensity of the signals and less formed TPs. A combination of 60% methanol and 20% of each ACN and water lead to good signal intensities of the TPs and reproducible results. 

**Figure 2:**
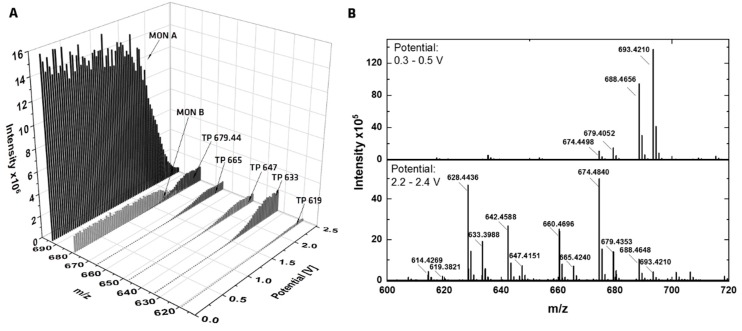
Online EC-MS measurements of MON. (**A**) Mass traces (*m/z*) of the transformation products of MON as sodium adducts in dependence of the applied oxidation potential ramped from 0–2.5 V (ramping rate: 20 mV/s. (**B**) Mass spectra of MON at a low potential of 0.3–0.5 V (upper spectrum) and at a higher potential of 2.2–2.4 V (lower spectrum).

In Figure 2A, a three-dimensional mass voltammogram of MON is plotted, presenting the mass spectra against the applied potential (0–2.5 V) using the GC working electrode and the optimized mobile phase. The signal intensities of MON A and MON B decrease with increasing potential, which corresponds to the signal intensities of five TPs. Related mass spectra are plotted in Figure 2B, the upper spectrum, with a low applied potential shows only MON A and MON B, both as protonated sodium adduct [M + Na]^+^ and as protonated ammonium adduct [M + NH_4_]^+^. The mass difference between the adducts is *m/z* 4.955. The second spectrum (lower one) shows MON A and B with low intensities and the five TPs are visible. All TPs were recorded as sodium and ammonium adducts, with ammonium adducts being more intense than sodium adducts.

**Figure 3:**
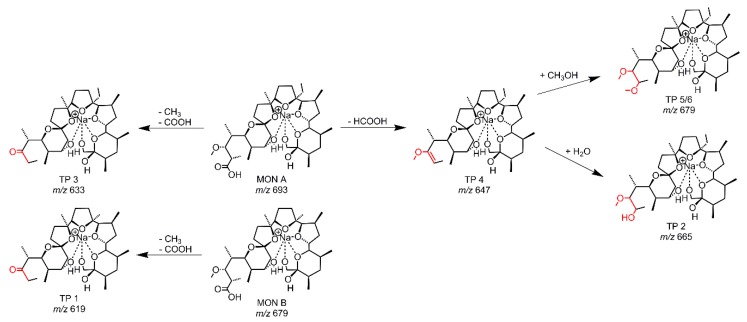
Proposed structures of the TPs derived from EC-experiments with MON A using a GC-electrode.

The HRMS data enables to determine the accurate mass of the TPs, followed by a calculation of a sum formula where suggestions of the transformations of MON become visible. The occurring transformations caused by applying an electrochemical potential are demethylations, decarboxylations with dehydrogenations and the addition of water or methanol (from solvent) to MON. A structural elucidation of the TPs is hard to make based on the accurate masses alone. For this reason, LC/HRMS measurements with MS/MS fragmentation were performed. After preparing an EC-reaction mix (GC-electrode, 0–2.5 V), the mix was separated using a RP-18 column. The chromatogram (Figure S1) showed six TPs next to MON A and MON B. There was one more TP compared to the online measurements, with two peaks showing the same accurate mass (TP 5 and TP 6) rendering them inseparable in direct infusion experiments. In Table 1, the EC-GC found TPs are listed together with the suggested transformation of MON A. In general, the TP can be formed from MON A or MON B, and for this determination, the MS/MS data is necessary. The ESI-MS fragmentation pathway of MON has been described in literature by Lopes et al. [31,32] and Sun et al. [23]. The whole fragmentation pathway is shown in Figure S2 [23,31,32]. There are different routes for fragmentation starting at the terminal positions (carboxy-group or tetrahydropyran-ring) of MON. The stable backbone consists of three tetrahydrofuran rings. Thus, the distinction whether a TP is formed from MON A or MON B will be apparent in all known fragments. Due to the different starting points of the fragmentation pathways, it is possible to localize the position/or side of the transformation. The recorded MS/MS data lead to the proposed structures of the TPs of the EC-GC reaction mix (Figure 3). The classification of the MS/MS-fragments to the transformation pathway is given in Tables S3 and S4. It could be shown, as a general result, that the transformation takes place at the carboxy-terminus of MON. TP 1 (*m/z* 619.382) is based on MON B and the same transformation occurs for TP 3 with *m/z* 633.397 based on MON A. Both TPs showed a demethylation and decarboxylation. The highest intensity was observed for TP 3. TP 4 results from a decarboxylation with dehydrogenation of MON A. The subsequent addition of water to the newly formed double bond leads to TP 2, whereas the addition of methanol leads to TP 5/6. As TP 5/6 has the same accurate mass but different retention times on a RP-18 column, stereoisomers could be assumed. Stereoisomers of TP 2 were not found, but the intensity of TP 2 is much lower than for TP 5/6, which is why only one TP with water addition was identified. The addition of methanol to substances by EC was described previously in studies on mycotoxins [33,34].

**Figure 4:**
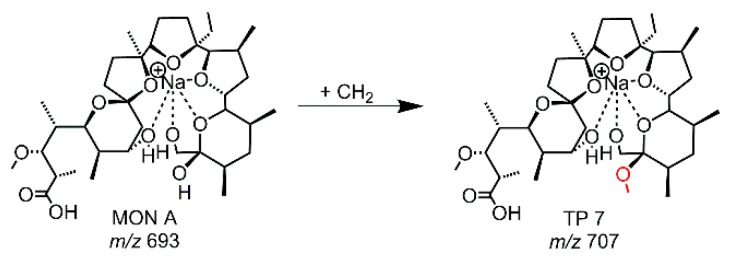
Proposed structure of TP 7 derived from EC-experiments with MON A using a MD-electrode.

While the use of a GC-electrode led to several different TPs shown in Figure 3, the electrochemical treatment of a MON-solution (containing methanol, ACN, water) using a MD-electrode revealed only one TP (TP 7, *m/z* 707.434). TP 7 is a methylation product of MON A (see Figure 4, Table S3). The evaluation of the MS/MS data pointed out that the methylation occurs at the tetrahydropyran-ring-terminus of MON. 

The obtained results reveal different reaction mechanisms of GC- and MD-electrodes leading to quite different TPs. The oxidation of MON occurs at the surface of the working electrode, which is why the electrode material has an influence on the formation of TPs. The GC working electrode consist of a nongraphitizable carbon material and the MD electrode is a boron-doped diamond electrode. MON is more oxidized with the glassy carbon electrode, although the maximum potential range of the MD-electrode is higher. 

### 2.2. Microsomal Tests

**Figure 5:**
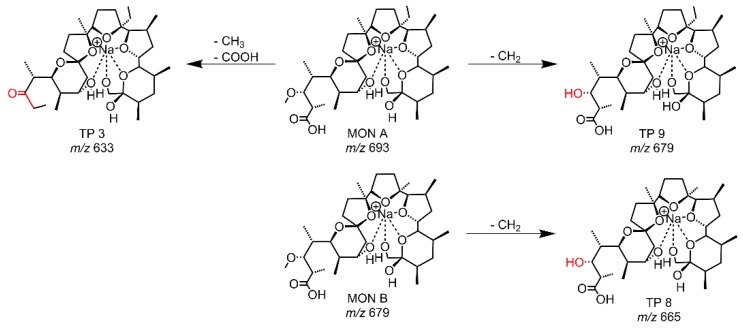
Proposed structures of the TPs resulting from RLM-experiments with MON A and MON B.

Metabolism tests with RLM were performed to identify and to compare the resulting metabolites with the TPs from the other simulation methods. In total, three TPs were observed as peaks of significant intensity besides MON A and MON B. As before, all structure proposals (see Figure 5, Table S5) were based on the accurate mass and the MS/MS data. TP 9 is generated from MON A and TP 8 from MON B, in both cases via O-demethylation, and TP 9 was previously described [17,18,19,20,21]. The third metabolite (*m/z* = 633.397 and RT = 109 s) had already been observed in the EC-LC/MS experiment as TP 3. The intensity of TP 3 produced by RLM was very low compared to the intensity of TP 3 generated by EC. 

Three metabolites of MON A were well known by literature and described as main metabolites [28], an *O*-demethylated one (*m/z* = 679), a hydroxylated one (*m/z* = 709) and an O-demethylated-hydroxylated one (*m/z* = 695). These metabolites were previously found by different studies investigating the biotransformation process of MON A [19,20,21], but not of MON B. Additionally, several other hydroxylated (*m/z* = 709) or oxidized (*m/z* = 707) TPs were known. The *O*-demethylated product was found as TP 9 of the RLM-tests, a hydroxylated TP could be observed in a very low intensity, which was too low to obtain MS/MS data. The metabolites were found in cattle and rat, and additionally, a metabolite with *m/z* 633 was found in steer liver and steer, calves, and rat feces. 

### 2.3. Hydrolysis of MON

**Figure 6:**
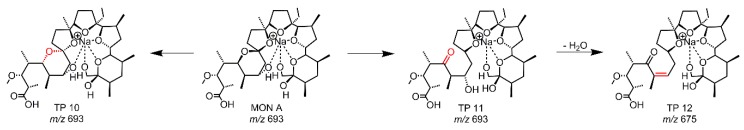
Proposed structures of the hydrolysis TPs of MON.

To identify hydrolysis products, MON was incubated with potassium biphthalate adjusted with acid or base to pH 3, pH 4, and pH 5. The samples were stored at 25 °C and measured continuously over 30 days as given in Table 2. At a pH of 3, the formation of TP 12 occurs after two days and at pH 4 after 4 days. TP 12 is not visible at a pH of 5 after 30 days, but TP 11 also occurs after 15 days. It is assumed that, after a longer incubation time, TP 12 will also be formed at pH 5. The structures of the TPs are shown in Figure 6 (Table S6). TP 10 and 11 have the same accurate mass as MON A and the MS/MS fragmentation showed similar fragments. TP 10 is a diastereomer of MON, due to a reversible acid-catalyzed reaction with racemization at the spiroketal. In a further reaction, the spiroketal is opened and TP 11 is formed. Due to the ring cleavage, the corresponding hydroxyl group is no longer capable for complexation. This effect enables the subsequent dehydration leading to TP 12. The reaction pathways forming TP 10 and TP 11 are strongly pH-dependent, a finding which is underpinned by the results shown in Table 2. Due to the acid-catalyzed reactions, the formation of TP 10 and TP 11 decrease with increasing pH-value. As TP 12 is derived in a subsequent reaction from TP 11, its formation kinetics are mainly determined by the incubation time. Thus, it is assumed that TP 12 will be detected after a longer storage time. In a previous study on the pH-dependent hydrolysis of MON by Sun et al. [26], three hydrolysis products of MON were characterized at pH 3. We also identified these three hydrolysis products (TP 10-12) and the acid-catalyzed formation pathway is described in more detail in Reference [26].

We found the known hydrolysis TPs of MON, but in relation to the previous study for higher pH values. All three TPs were occurring at pH 3 and 4 and the process is already started at pH 5. It is assumed that TP 12 will be formed at pH 5 by extended reaction time. The hydrolysis of MON has an influence on the degradation of MON. By longer storages of the EC-reaction mixes, hydrolysis TP 11 was first found (data not shown). 

## 3. Materials and Methods 

### 3.1. Chemicals

Monensin sodium salt (purity 85%, VetranalTM) was purchased from Sigma-Aldrich (Steinheim, Germany). KH_2_PO_4_ were purchased from Chemsolute (Renningen, Germany) and K_2_HPO_4_ from Carl Roth (Karlsruhe, Germany). NADPH tetrasodium salt was obtained from Carl Roth (Karlsruhe, Germany). Potassium biphthalate was obtained by Fluka Chemika (Buchs, Switzerland).

Acetonitrile and methanol were purchased from Chemsolute (Renningen, Germany). Ammonium formate from Fluka Chemika (Buchs, Switzerland) and formic acid from Merck (Darmstadt, Germany). Hydrochloric acid was obtained by Chemsolute (Renningen, Germany) and sodium hydroxide by Merck (Darmstadt, Germany). Ultrapure water was produced by a Purelab Flex 2 system, ELGA Veolia Water technologies (Celle, Germany). All standard chemicals were of p.a. grade and all solvents (acetonitrile, methanol) of LC/MS grade.

### 3.2. Microsomal Sources and Incubation

Rat liver microsomes (RLM) were purchased from Thermo Fisher Scientific (Pittsburgh, PA, USA). The RLM were prepared from Sorague Dawley male rats with a protein concentration of 20 mg/mL and a CYP450 content of 319 pmol/mg protein. The total protein content and CYP450 concentrations were provided by the manufacturer.

Incubations with RLM were carried out in a volume of 200 µL. The mixture contained 6.25 µmol/L MON dissolved in ACN, microsomes (1.0 mg/mL microsomal protein), 0.1 M potassium phosphate buffer (pH 7.4), and 0.01 mM MgCl_2_. The fraction of ACN in the incubation mixture was not higher than 3%. After pre-incubation of 5 min at 37 °C, 0.6 mM NADPH was added to the mixture to start the enzymatic reaction (incubation time: 90 min, 37 °C, 800 rpm). To stop the reaction, 50 µL ACN (−20 °C) was added and the sample was mixed thoroughly for 30 s. The incubation mixture was centrifuged at 12 rpm. The supernatant was analyzed by LC/HRMS (Agilent Technologies, Waldbronn, Germany/Sciex, Darmstadt, Germany). Control incubations, where the amount of NADPH was replaced through potassium buffer, were performed in duplicate. The reaction was performed in triplicate.

### 3.3. EC/ESI-HRMS

The electrochemical oxidation was performed with the ROXY^TM^ system (Antec Scientific, Zoeterwoude, The Netherlands) containing potentiostat and electrochemical flow-through cell. The instrument was controlled via Dialogue Elite software (Antec Leyden, Zoeterwoude, The Netherlands) version 2.0.0.81. Mass voltammograms were recorded by online coupling of the electrochemical cell to an electrospray ionization source of a TripleTOF^®^ 6600 Quadrupole Time-Of-Flight (QTOF) mass analyzer (Sciex, Darmstadt, Germany) controlled via Analyst^®^ TF1.8.0 including data processing. The software Origin 2018 (OriginLab, Northampton, MA, USA) was used for the graphical representation of the three-dimensional mass voltammograms. The MON-solvent-mixture consisting of 20 µM MON in methanol:acetonitrile:water (3:1:1; *v/v/v*) with 1 mM ammonium formiate, were passed through the cell by a Legato^®^ 110 dual rate system syringe pump (KD Scientific, Hollison, MA USA) with a flow rate of 30 µL/min. The EC cell consists of a three-electrode arrangement including a titanium auxiliary electrode (inlet-block of the cell), a HyREF^TM^-reference electrode (Pd/H_2_), and a working electrode. Two different working electrode materials were used, a glassy carbon (GC) or a boron-doped diamond (MD) electrode. The applied potential by the potentiostat was dependent of the working electrode material, for the GC electrode a potential ramped between 0 and 2.5 V and for the MD between 0 and 3.5 V was applied, with a scan rate of 20 mV/s. The working electrode was activated before each measurement by a manufacturer-provided pulse cleaning program. The EC-cell was connected to the ESI-HRMS. The experiment parameters for MS-detection were given in Table 3. Each mass voltammogram was recorded at least three times to ensure the reproducibility of the measurements. Control measurements were performed by using the solvent without analyte. Next to the online measurements, aliquots were collected offline into a HPLC-vial from the EC-cell and were used for further LC/HRMS measurements. 

### 3.4. Hydrolysis

For the hydrolysis experiments pH-buffers after *Clark and Lubs* [16] were prepared with a pH of 3, 4, and 5. The buffer mixtures are given in Table 4. The pH values were measured with a pH-meter CG 837 (Schott, Mainz, Germany), directly before starting the experiment. The initiation was started by spiking the buffer with MON (1 µM) in a vial. The samples were shaken for 30 s and afterwards stored at 23 °C. Each sample was performed in triplicate. All samples were measured with the LC/HRMS at defined dates.

### 3.5. LC/HRMS 

All products from electrochemical-, microsomal-tests, and hydrolysis were analyzed by LC/HRMS using TripleTOF^®^ 6600 Quadrupole Time-Of-Flight (QTOF) mass analyzer (Sciex, Darmstadt, Germany) connected to an Agilent 1290 Infinity II (Agilent Technologies, Waldbronn, Germany), consisting of a 1290 Infinity II multisampler, a 1290 Infinity II flexible pump, a 1260 Infinity II diode array detector HS, and 1290 Infinity II multicolumn thermostat. The system was controlled via Analyst^®^ TF1.8.0 (AB Sciex) and the data were processed by SciexOS and using in-house scripts of the statistical working environment R (REF). The analytical column was a ZorbaxEclipse Plus C18, particle size 1.8 µm, 50 × 2 mm (Agilent Technologies, Waldbronn, Germany) and the column oven was set to 25 °C. For the separation of the different samples of MON and its TPs, a mobile phase consisting of (A) H_2_O + 0.1% formic acid and (B) ACN + 0.1% formic acid was used. The injection volume was 2 µL. The flow rate of the mobile phase was 0.8 mL/min, and a gradient program was used for the separation. Starting was 50% of B, within 0.5 min it was raised to 90%. After 3.5 min it was decreased to 50% and the column was re-calibrated for 3 min. The conditions for the TTOF are listed in Table 5, an information dependent acquisition (IDA) was included for MS/MS experiments. To assign a potential sum formula and chemical structure to a measured ion mass, we calculated all possible sum formulas within 3 ppm deviation around the respective *m/z*, allowing only the elements C, H, and O, together with Na and N, to account for adduct formation. The mass accuracy of Sciex TTOF is < 2 ppm and was confirmed via a tuning run before any measurement. Whenever more than one suggestion remained, we used a fit of the isotopic peak distribution (Sigma value) to additionally rank candidates and select the best suggestion. MS/MS spectra, which were acquired in IDA mode, allowed a further inference of structural confirmation of the precursor molecule.

### 3.6. EC/ESI-MS—LC/MS

The measurements to find the optimized parameters for the EC investigations (Table S1) were done by a ROXY^TM^ system (Antec Scientific, Zoeterwoude, The Netherlands) containing potentiostat and electrochemical flow-through cell. The instrument was controlled via Dialogue software (Antec Leyden, Zoeterwoude, The Netherlands) version 2.02.199. The EC-cell was coupled to an electrospray ionization source of a single quadrupole mass spectrometer (Agilent Technologies GmbH, Waldbronn, Germany). The optimization of the gradient for LC separation were performed with an Agilent 1290 Infinity (Agilent Technologies, Waldbronn, Germany) UPLC-system, consisting of a 1290 Infinity Sampler, a 1290 Infinity quat. pump, a 1260 Infinity diode array detector, and 1290 Infinity TCC. The system was controlled via OpenLab CDS. The analytical column was a ZorbaxEclipse Plus C18, particle size 1.8 µm, 50 × 2.1 mm (Agilent Technologies, Waldbronn, Germany). 

## 4. Conclusions

This study extensively investigated the generation of TPs from Monensin by three different simulation methods: Electrochemical oxidation, metabolism tests with microsomes, and hydrolysis. The major mechanisms of MON transformation are decarboxylation, O-demethylation and acid-catalyzed ring-opening reactions. For all found TPs, structures were proposed based on the accurate masses and additional MS/MS spectra. All TPs showed shorter retention times on revered-phase chromatography compared to the parent substance MON A, indicating their higher polarity. Most TPs (six) were found by electrochemical investigations using a GC electrode and one TP by using MD-electrode. Three individual TPs were found for the microsomal assay and the hydrolysis study, respectively. In total, twelve different TPs were found, subdivided to two TPs of MON B and ten TPs of MON A. In general, nine structurally different TPs were identified, whereby four transformations are described for the first time, all generated with EC. TP 3, being commonly found in microsome and EC studies, confirmed the potential of EC to simulate biotransformation processes of drugs.

## Figures and Tables

**Table 1:** HRMS data of TPs as sodium adducts of MON. The TP intensity is calculated in relation to MON A (strong–s: 60–100%, moderately strong–ms: 30–60%, weak–w: 10–30%, very weak–vw: < 10%).

TP Simulation Method	Compound	Retention Time [s]	calc. *m/z*	Molecular Formula	Suggested Transformation	TP Intensity
Std	MON A	162	693.4184	C_36_H_62_O_11_Na	-	s
Std	MON B	120	679.4028	C_35_H_60_O_11_Na	-	w
EC-GC	TP 1	92	619.3817	C_33_H_56_O_9_Na	-CO_2_ -2x CH_2_	w
EC-GC	TP 2	102	665.4235	C_35_H_62_O_10_Na	-CO_2_ -2H +H_2_O	w
EC-GC, RLM	TP 3	109	633.3973	C_34_H_58_O_9_Na	-CO_2_ -CH_2_	s
EC-GC	TP 4	132	647.4130	C_35_H_60_O_9_Na	-CO_2_ -2H	ms
EC-GC	TP 5	133	679.4392	C_36_H_64_O_10_Na	-CO_2_ -2H +OCH_3_ +H	s
EC-GC	TP 6	144	679.4392	C_36_H_64_O_10_Na	-CO_2_ -2H +OCH_3_ +H	ms
EC-MD	TP 7	107	707.4341	C_37_H_64_O_11_Na	+CH_2_	vw
RLM	TP 8	79	665.3877	C_34_H_58_O_11_Na	-2 CH_2_	vw
RLM	TP 9	95	679.4028	C_35_H_60_O_11_Na	-CH_2_	w
Hyd	TP 10	75	693.4184	C_36_H_62_O_11_Na	diastereomer	s
Hyd	TP 11	99	693.4184	C_36_H_62_O_11_Na	ring cleavage	ms
Hyd	TP 12	86	675.4079	C_36_H_60_O_10_Na	-H_2_O	s

**Table 2:** Formation of hydrolysis TPs 10–13 of MON over a storage period of 30 days at pH 3, 4, and 5 at a temperature of 25 °C. The ‘x’ indicates the occurrence of the peak in the MS-spectrum. MON represents MON A and MON B.

	Day							
pH 3	1	2	3	4	5	8	15	30
MON	x	x	x	x	x	x	x	x
TP10	x	x	x	x	x	x	x	x
TP11	x	x	x	x	x	x	x	x
TP12		x	x	x	x	x	x	x
	**Day**							
**pH 4**	**1**	**2**	**3**	**4**	**5**	**8**	**15**	**30**
MON	x	x	x	x	x	x	x	x
TP10		x	x	x	x	x	x	x
TP11			x	x	x	x	x	x
TP12				x	x	x	x	x
	**Day**							
**pH 5**	**1**	**2**	**3**	**4**	**5**	**8**	**15**	**30**
MON	x	x	x	x	x	x	x	x
TP10				x	x	x	x	x
TP11							x	x
TP12								

**Table 3:** Parameters of the ESI-Triple TOF^®^ for the EC/MS measurements.

Experiments Parameters		Mass Range Parameters	
gas temperature	350 °C	collision energy	10 V
ion source gas 1 (nitrogen)	20 L/min	declustering potential	80 V
ion source gas 2 (nitrogen	15 L/min	mass range	200–1000 Da
curtain gas (nitrogen)	25 L/min		
ion spray voltage floating	+5500 V		

**Table 4:** Buffer mixtures after *Clark and Lubs*, the volume of each is filled up with water to 10 mL.

	V Potassium Biphthalate Buffer–0.1 M [mL]	V Hydrochloric Acid–0.1 M [mL]	V Sodium Hydroxide–0.1 M [mL]	pH Measured
pH 3	5	2.032	-	3.06
pH 4	5	-	0.04	4.02
pH 5	5	-	2.385	5.04

**Table 5:** Parameters of the ESI- Triple TOF^®^ for the LC/HRMS measurements.

Experiments Parameters		Mass Range Parameters	
gas temperature	400 °C	MS 1	
ion source gas 1 (nitrogen)	50 L/min	collision energy	10 V
ion source gas 2 (nitrogen	55 L/min	declustering potential	80 V
curtain gas (nitrogen)	45 L/min	mass range	100–800 Da
ion spray voltage floating	+5500 V	MS2	
		collision energy	85 V
		collision energy spread	20 V
		declustering potential	80 V
		mass range	50–800 Da

## Supplementary Materials

The following are available online at, Table S1: Overview of tested EC-conditions; Figure S1: EIC-chromatograms of all MON-samples; Figure S2: ESI-fragmentation pathway, Tables S2–S6: classification of measured TPs to known fragments of S2: MON A and B; S3 and S4: EC-GC and EC-MD; S5: RLM-tests, S6: hydrolysis.
