# Supplementary material for: Prediction of Transformation Products of Monensin by Electrochemistry Compared to Microsomal Assay and Hydrolysis
Source: Molecules. 2019 Jul 27;24(15):2732. doi: 10.3390/molecules24152732 (PMC6696283; doi:10.3390/molecules24152732)
Supplement: Supplementary file 1 [file molecules-24-02732-s001.pdf]

molecules

Article

Supplementary Materials

Table S1: Optimization of the EC-MS measurements, given are tested combinations of electrode, solvents, modifier and potential polarity. (GC: glassy carbon; MD: magic diamond; MeOH: methanol; ACN: acetonitrile; AF: ammonium formiate; FA: formic acid).

electrode	solvent (v/v)	modifier	potential polarity
GC, MD	MeOH:H_2O (2:1)	AF (1 mM),<br>FA (0,1%)	pos
GC, MD	ACN:H_2O (2:1)	AF (1 mM),<br>FA (0,1%)	pos
GC, MD	MeOH:ACN:H_2O<br>(1:3:1) (1:1:1)	AF (1 mM)	pos
GC	MeOH:ACN:H_2O (3:1:1)	AF (1 mM,<br>2 mM, 5 mM)	pos, neg
MD	MeOH:ACN:H_2O (3:1:1)	AF (1 mM)	pos, neg

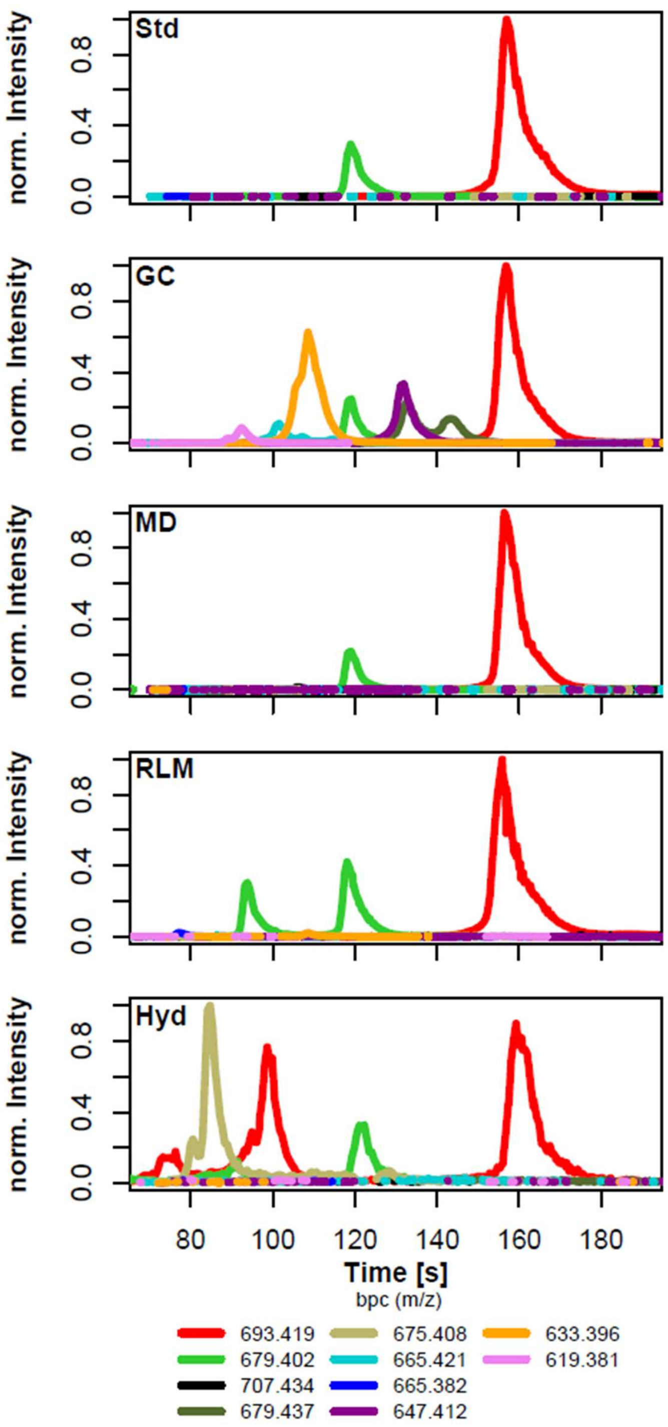

Figure S1: Extracted-Ion-Chromatogram (+ESI, LC-HRMS) of different investigated samples of MON. The first chromatogram shows the used standard of MON, the second chromatogram the results of the EC-GC reaction mix, the third one the reaction mix of EC-MD. Then the chromatogram of the RLM-test followed and last the hydrolysis sample (pH 3).

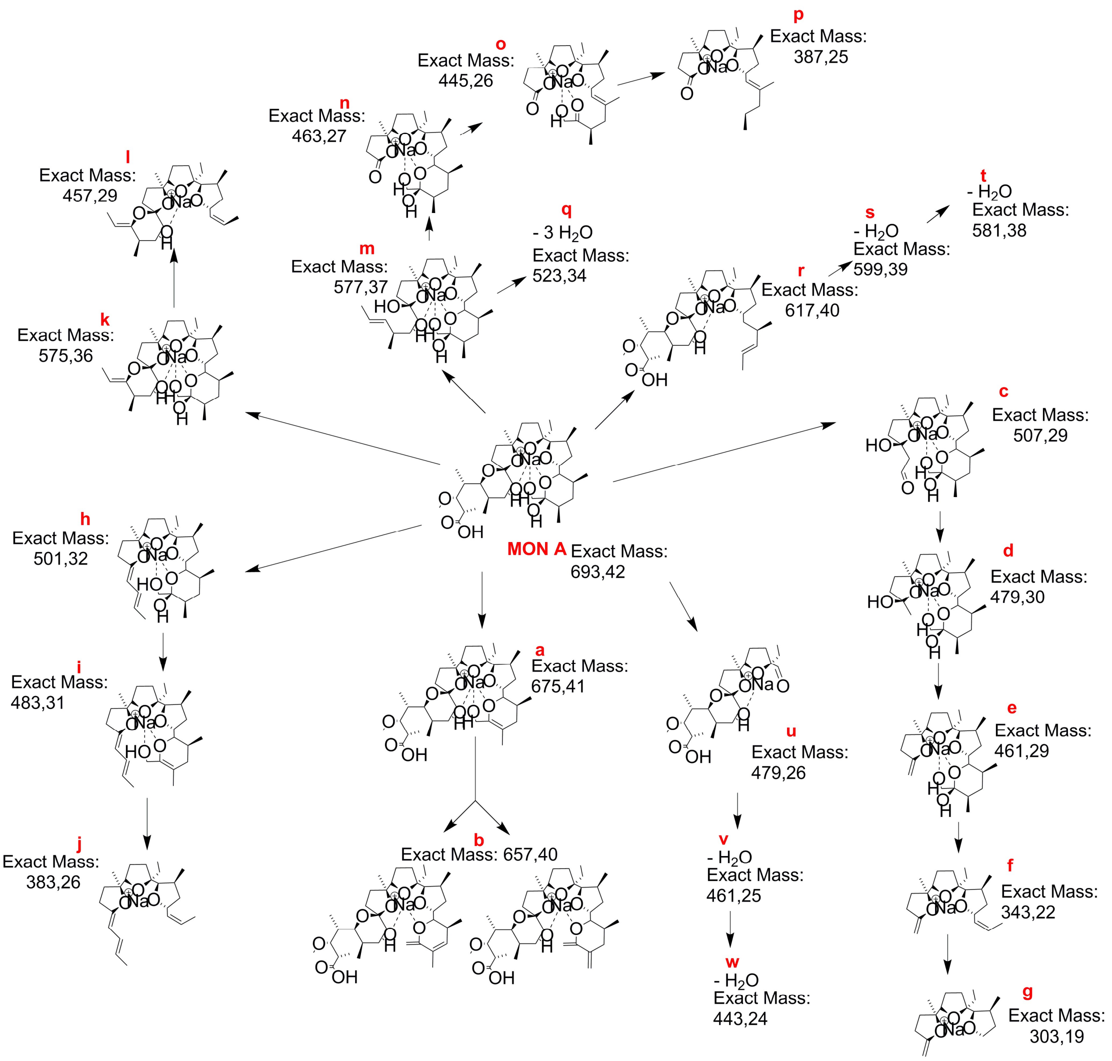

Figure S2: Literature known ESI-MS fragmentation pathway of MON. Summary of the studies of Lopes et. al, [1,2] Fragments a-p and Sun et. al [3] Fragments r-w.

Table S2: Fragment scheme of fragment ions observed in the MSMS/Spectra of MON A and MON B. The height is calculated to the max. intensity of the highest fragment.

name	MON A			MON B	
sum formula	$C_{36}H_{62}O_{11}Na$			$C_{35}H_{60}O_{11}Na$	
transformation	-			- (-CH_2)	
Fragment #	m/z	height	#	m/z	height
	693.4162	27		679.4000	27
a	675.4051	29	a	661.3874	24
b	657.3928	6	b	643.3761	6
c	507.2901	2	c	493.2714	3
d	479.2961	89	d	465.2788	100
e	461.2850	100	e	447.2686	82
f	343.2192	1	f	-	
g	303.1906	2	g	289.1734	3
h	501.3150	25	h	487.3007	24
i	483.3050	7	i	469.2888	9
j	383.2507	1	j	369.2366	2
k	575.3531	1	k	561.3334	1
l	-		l	-	
m	577.3678	2	m	563.3488	4
n	463.2619	1	n	449.2497	3
o	445.2526	1	o	431.2384	2
p	387.2465	1	p	373.2306	3
q	523.3358	1	q	509.3202	2
r	617.3995	3	r	603.3811	6
s	599.3886	15	s	585.3732	14
t	581.3794	16	t	567.3620	20
w	443.2753	12	w	429.2580	14

Table S3: Fragment scheme of fragment ions observed in the MSMS/Spectra of EC-generated TP 1 to TP3. The height is calculated to the max. intensity of the highest fragment.

name	*GC* TP 1			*GC* TP 2			*GC* TP 3	
sum formula	$C_{33}H_{56}O_9Na$			$C_{35}H_{62}O_{10}Na$			$C_{34}H_{58}O_9Na$	
transformation	- $C_2H_4O_2$			- CO			- $C_2H_4O_2$	
Fragment #	m/z	height	#	m/z	height	#	m/z	height
	619.3600	100		665.4007	100		633.3757	100
a	601.3512	52	a	647.3905	88	a	615.3654	44
b	583.3388	19	b	629.3784	22	b	597.3548	7
c-B	493.2587	27	c-A	507.2733	30	c-A	507.2747	19
d	465.2663	22	d-A	479.2794	25	d-A	479.2808	12
e-B	447.2553	35	e-A	461.2705	30	e-A	461.2705	19
f	-	-	f	-	-	f	-	-
g-B	289.1693	8	g-A	303.1789	7	g-A	303.1807	2
h-B	487.2802	18	h-A	501.3012	32	h-A	501.2993	10
i-B	469.2733	17	i-A	483.2894	14	i-A	483.2907	4
j	-	-	j	-	-	j	-	-
k	-	-	k	-	-	k	-	-
l	-	-	l	-	-	l	-	-
m	-	-	m	-	-	m	-	-
n-B	449.2318	8	n-A	463.2479	4	n	463.2503	2
o	-	-	o-A	445.2387	4	o-A	445.2385	1
p-B	373.2218	5	p-A	387.2326	5	p-A	387.2323	1
q	-	-	q	-	-	q	-	-
r	-	-	r	-	-	r	557.3228	1
s	-	-	s	-	-	s	-	-
t	-	-	t	-	-	t	-	-
w-B	429.2461	16	w-A	443.2594	13	w-A	443.2607	5

Table S4: Fragment scheme of fragment ions observed in the MSMS/Spectra of EC-generated TP 4 to TP6 and TP7 (EC-MD). The height is calculated to the max. intensity of the highest fragment.

name	*GC* TP 4		*GC*	TP 5		*GC*	TP 6		*MD*	TP 7	
sum formula	$C_{35}H_{60}O_9Na$			$C_{34}H_{58}O_9Na$			$C_{34}H_{58}O_9Na$			$C_{36}H_{62}O_{12}Na$	
transformation	- CH_2O_2			- $C_2H_4O_2$			- $C_2H_4O_2$			$+CH_2$	
Fragment #	m/z	height		m/z	height		m/z	height		m/z	height
	647.3910	100		679.4155	99		679.4155	96		707.4112	7
a	629.3804	76	a	661.4049	100	a	661.4048	100	a	689.3935	4
									a-A	675.3845	70
b	611.3713	16	b	643.3949	27	b	643.3956	25	b-A	657.3749	26
c-A	507.2748	51	c-A	507.2757	41	c-A	507.2731	40	c	-	-
d-A	479.2805	30	d-A	479.2792	28	d-A	479.2802	28	d	493.2967	11
e-A	461.2700	42	e-A	461.2703	34	e-A	461.2702	36	e-A	461.2684	100
f	-	-	f	-	-	f	-	-	f-A	343.2114	9
g-A	303.1811	7	g-A	303.1798	5	g-A	303.1819	5	g-A	303.1823	12
h-A	501.3005	43	h-A	501.3004	43	h-A	501.2992	37	h	515.2994	4
i-A	483.2901	22	i-A	483.2912	20	i-A	483.2892	20	i-A	483.2914	44
j	-	-	j	-	-	j	-	-	j-A	383.2402	11
k	-	-	k	-	-	k	-	-	k-A	575.3329	9
l	-	-	l	-	-	l	-	-	l	471.2893	4
m	-	-	m	-	-	m	-	-	m	-	-
n-A	463.2478	9	n-A	463.2503	6	n-A	463.2493	5	n-A	463.2931	5
o-A	445.2404	6	o-A	445.2404	5	o-A	445.2416	3	o-A	445.2416	5
p-A	387.2367	5	p-A	387.2362	5	p-A	387.2365	5	p	401.2448	4
/			/			/			p-A	387.2353	19
q	-	-	q	-	-	q	-	-	q	-	-
r-A	617.3802	19	r	-	-	r	-	-	r	-	-
s-A	599.3703	29	s	-	-	s	-	-	s	613.3959	4
/			/			/			s-A	599.3660	13
t-A	581.3594	10	t	567.3457	3	t	-	-	t	581.3581	31
w	-	-	w-A	443.2608	11	w-A	443.2595	10	w-A	443.2612	73

Table S5: Fragment scheme of fragment ions observed in the MSMS/Spectra of the TPs of the RLM-experiments. The height is calculated to the max. intensity of the highest fragment.

name	*RLM* TP 8			*RLM* TP 9			*RLM* TP 3	
sum formula	$C_{35}H_{62}O_{10}Na$			$C_{34}H_{58}O_{11}Na$			$C_{34}H_{58}O_{9}Na$	
transformation	- CH_2			- 2 CH_2			- $C_2H_4O_2$	
Fragment #	m/z	height	#	m/z	height	#	m/z	height
	679.3980	27		665.3832	47		633.3915	100
a	661.3875	25	a	647.3673	40	a	615.3824	57
b	643.3765	8	b	629.3623	10	b	597.3708	16
c-A	507.2881	4	c-B	493.2742	19	c-A	507.2880	32
d-A	479.2948	45	d-B	465.2787	62	d-A	479.2929	26
e-A	461.2841	100	e-B	447.2654	100	e-A	461.2823	26
f-A	343.2228	2	f-B	329.2041	5	f	-	-
g-A	303.1894	2	g-B	289.1672	5	g-A	303.1979	5
h-A	501.3148	38	h-B	487.2971	43	h-A	501.3129	24
i-A	483.3048	10	i-B	469.2884	14	i-A	483.3058	13
j-A	383.2461	1	j	-	-	j	-	-
k	-	-	k	-	-	k	-	-
l	-	-	l	-	-	l	-	-
m-A	577.3684	2	m	549.3394	5	m	-	-
/			m-B	563.3339	14	/		
n-A	463.2632	2	n-B	449.2523	10	n-A	463.2684	5
o-A	445.2502	2	o	-	-	o-A	445.2601	5
p-A	387.2471	4	p-B	373.2381	10	p-A	387.2445	5
q-A	523.3332	3	q-B	509.3213	19	q	-	-
r-A	617.3959	6	r	589.4793	5	r	-	-
/			r-B	603.3751	10	/		
s-A	599.3876	8	s-B	585.3731	19	s	-	-
t-A	581.3759	7	t-B	567.3560	24	t	-	-
w	-	-	w-B	429.2556	34	w-A	443.2711	17

Table S6: Fragment scheme of fragment ions observed in the MSMS/Spectra MSMS/Spectra of the hydrolysis products TP 10 to TP 12. The collision energy of experiments was set to 40 V. The height is calculated to the max. intensity of the highest fragment (a-w).

name	*hyd* TP 10		*hyd*	TP 11		*hyd*	TP 12	
sum formula	$C_{36}H_{62}O_{11}Na$			$C_{36}H_{60}O_{10}Na$			$C_{36}H_{62}O_{11}Na$	
transformation	-			- H_2O				
Fragment #	m/z	height	#	m/z	height	#	m/z	height
	693.4203	3056		693.4188	813		675.4092	4805
a	675.4086	56	a	675.4055	23	a	657.3977	100
b	657.4167	5	b	657.4074	9	b	639.3913	9
c	507.2735	2	c	-	-	c	-	-
d	479.2995	94	d	479.2970	100	d	-	-
e	461.2861	100	e	461.2861	43	e	-	-
f	-	-	f	-	-	f	-	-
g	-	-	g	-	-	g	-	-
h	501.3282	5	h	501.3069	9	h	-	-
i	483.3126	2	i	-	-	i	-	-
j	-	-	j	-	-	j	-	-
k	-	-	k	-	-	k	-	-
l	-	-	l	-	-	l	-	-
m	577.3895	5	m	577.3722	4	m	559.3599	12
n	-	-	n	-	-	n	-	-
o	-	-	o	-	-	o	-	-
p	387.2603	2	p	-	-	p	-	-
q	-	-	q	-	-	q	-	-
r	617.4222	2	r	617.4130	4	r	599.3870	19
s	599.4028	2	s	599.3940	4	s	581.3748	16
t	581.3899	3	t	581.3744	4	t	-	-
w	-	-	w	-	-	w	-	-

molecules

Supplementary Materials: The following are available online at www.mdpi.com/xxx/s1. Figure S1: ESI-fragmentation pathway. Table S1-S5: classification of measured TPs to known fragments of S1: MON A and B; S2 and S3: EC-GC and EC-MD; S4: RLM-tests. S5: hydrolysis.

Sample Availability: Samples of the compounds ...... are available from the authors.

© 2019 by the authors. Submitted for possible open access publication under the terms and conditions of the Creative Commons Attribution (CC BY) license (http://creativecommons.org/licenses/by/4.0/).

References

1. Lopes, N.P.; Stark, C.B.; Hong, H.; Gates, P.J.; Staunton, J. Fragmentation studies on monensin A and B by accurate-mass electrospray tandem mass spectrometry. *Rapid Commun Mass Spectrom* **2002**, *16*, 414-420, doi:10.1002/rcm.592.
2. Lopes, N.P.; Stark, C.B.W.; Gates, P.J.; Staunton, J. Fragmentation studies on monensin A by sequential electrospray mass spectrometry. *Analyst* **2002**, *127*, 503-506, doi:10.1039/b110412h.
3. Sun, P.; Cabrera, M.L.; Huang, C.H.; Pavlostathis, S.G. Biodegradation of veterinary ionophore antibiotics in broiler litter and soil microcosms. *Environ Sci Technol* **2014**, *48*, 2724-2731, doi:10.1021/es404619q.
